# Magnitude of overweight and associated factors among type 2 diabetes mellitus patients at Mekelle public hospitals, Tigray, Ethiopia: a cross-sectional study

**DOI:** 10.1186/s13104-019-4791-1

**Published:** 2019-11-21

**Authors:** Kbrom Gemechu Kiros, Gebre Yitayih Abyu, Desta Siyoum Belay, Mekonnen Haftom Goyteom, Tensay Kahsay Welegebriel

**Affiliations:** 1 Department of Nursing, Adigrat University, Adigrat, Ethiopia; 2 Department of Nursing, Bahirdar University, Bahir Dar, Ethiopia; 3 Department of Nursing, Mekelle University, Mekelle, Ethiopia

**Keywords:** Overweight, Determinant factors, Type 2 diabetes mellitus

## Abstract

**Objective:**

To assess magnitude of overweight and associated factors among type 2 diabetes mellitus patients at Mekelle public hospitals, Tigray, Ethiopia.

**Results:**

A total of 365 participants were enrolled in this study. One hundred ninety-eight (54.2%) of the participants were males and 288 (78.9%) of the study participants were from an urban residence. In this study 161 (44.1%) and 12 (3.3%) of the study subjects were alcohol consumers and smokers respectively. Besides, 166 (45%) of the study participants had poor dietary intake and around 302 (82.7%) had low level of vigorous physical activity. The proportion of individuals who were overweight using body mass index as a measure was 149 (40.8%) and the proportion of individuals who had central obesity using waist circumference as a measure was 194 (53.2%). The magnitude of overweight among study participants from urban residence and alcohol consumers was 138 (92.6%) and 93 (62.4%) respectively. Residence area, alcohol consumption, physical activities, central obesity and dietary intake were the determinant factors for overweight among type 2 diabetes mellitus patients.

## Introduction

Globally, more than 1.9 billion adults aged 18 years and above were overweight in 2016. Of these, over 650 million adults were obese [1]. In Sub-Saharan African (SSA) countries the magnitude of overweight is increasing at an alarming rate [2]. This is due to rapid urbanization, dramatic lifestyle changes and high prevalence of child hood stunting [3]. Overweight has been an issue in developed countries for the past years. However, currently, it has gained attention in developing countries as an issue that needs to be addressed [4]. Overweight in people with diabetes could induce increased thrombogenic factors, cardiovascular disease and raised blood pressure. It also interferes with the treatment of hyperglycemia and diabetes-related complications [5, 6].

In Ethiopia, magnitude of overweight is increasing among type 2 DM patients. However, data on overweight of type 2 DM patients is limited because priority has always been given to under nutrition and communicable diseases [7, 8]. Besides, previous studies predominantly relied on body mass index (BMI) as measure of overweight. However, our study used both BMI and waist circumference to measure overweight. Therefore, the aim of this study was to assess magnitude of overweight and associated factors among type 2 DM patients.

## Main text

### Study area, design, period and participants

The study was conducted from February to April, 2018 by using cross-sectional study design in Mekelle public hospitals, Tigray, Ethiopia. All type 2 DM patients who were available at the time of data collection period were included and patients who had severe illness or physical disability, pregnant mothers and patients with edema were excluded from this study.

### Sample size determination and sampling technique

A single population proportion formula was used. The estimated proportion of overweight among type 2 DM patients was 31.5% [8]. Accordingly, the required sample size (n) was estimated with a confidence level of 95%, 5% margin of error and by adding 10% non-response rate the final sample size was 365.

Systematic random sampling method was used to select the study participants from a total of 2442 type 2 DM patients who were on treatment follow up in Mekelle public hospitals. To select the required sample size the total sample size was proportionally allocated to the three public hospitals. Accordingly, the list of the patients was taken from the follow up unit of the three public hospitals and sampling frame was developed. Then the first study subject was randomly selected from the sampling frame by using lottery method and based on the sampling interval (k = 6) every six interval was selected from the sampling frame. Finally, 365 of patients with type 2 DM were included in our study.

### Data collection tools and procedures

The tools for data collection include a portable Stadiometer, stretch-resistant tape meter and structured questionnaire. The questionnaire was composed of questions on socio-demographic data, behavioral and health-related factors, dietary factors and anthropometric measurement (weight, height and waist circumference).

The data was collected using a structured questionnaire through face to face interview and physical measurements of weight, height, waist circumferences using standardized techniques and calibrated equipment. Weight and height were measured with participants standing without shoes and wearing light clothing. Participants were standing upright with the head, shoulder, buttock, lower limb and heal of the foot touches the height board for height measurement. Waist circumference was measured midway between the lower rib margin and the iliac crest in the horizontal plane using a tape meter by following the standard procedure.

### Data quality control

The questionnaire was initially prepared in English and translated to Tigrigna language then back to the English language. One day training was given on the objective of the study, instrument and data collection procedures by the principal investigator for the data collectors and supervisors. The weight measurement scale was checked if it is at zero before each measurement. Five percent of the questionnaire was tested before the actual data collection period outside of the study area. Data collectors were instructed to check the completeness of the instrument just after its completion. The principal investigator checked out the questionnaire for completeness each night. Moreover, the collected data were coded, cleaned and explored before analysis to check missing items and completeness of the collected data.

### Operational definitions

*Overweight* BMI greater than or equals to 25 kg/m^2^.

*Central obesity* waist circumference greater than 88 cm for females and greater than 102 cm for males was considered as having abdominal obesity.

*Low level of physical exercise* individual activity less than 150 min per week was considered as low level of physical activities.

*Adequate level of physical exercise* individual activity above 150 min per week was considered as adequate level of physical activities.

*Dietary intake* level of dietary intake was determined based on dietary factors questionnaire. Six items were asked and based on the mean value of those questions individuals who score below the mean value were classified as poor and those who score above the mean value were classified as good dietary practices.

### Data processing and analysis

The collected data were entered and cleaned using Epi-data manager. Two items of dietary questions were reversely coded to explain total score to be interpreted as higher scores meaning better outcomes. Then it was exported to SPSS version 21 for statistical analysis. Descriptive statistics were computed using the frequency table and numerical summary measures. Binary logistic regression was done to determine the magnitude, direction and strength of association between a set of independent variables and the outcome variable at p < 0.25 significance level. Then those variables that were significant at p < 0.25 with the outcome variable were selected for multivariable analysis. Odds ratio with 95% confidence level was computed and significant association was declared at p-value < 0.05. Finally, the result was presented using text and tables.

## Results

### Socio-demographic characteristics

A total of 365 participants were enrolled in this study. One hundred ninety-eight (54.2%) of the participants were males. Two hundred seventy (74%) of the study subjects were Orthodox followers. Besides, 288 (78.9%) of the study participants were from an urban setting and 291 (79.7%) were married (Table 1).

**Table 1:** Socio-demographic characteristics of type 2 diabetes mellitus patients at Mekelle public hospitals, Tigray, Ethiopia, 2018

Variables	Number	Percent
Sex		
Male	198	54.2
Female	167	45.8
Age		
25–34	30	8.2
35–44	71	19.5
45–54	133	36.4
Above 55 years	131	35.9
Marital status		
Single	12	3.3
Married	291	79.7
Divorced	25	6.8
Widowed	37	10.2
Religion		
Orthodox	270	74
Muslim	78	21.4
Others	17	4.6
Ethnicity		
Tigray	354	97
Amhara	11	3
Educational status		
Can’t read and write	74	20.3
Primary	94	25.8
Secondary	72	19.7
Diploma and above	125	34.2
Occupational status		
Housewife	87	23.8
Government employee	127	34.8
Merchant	83	22.7
Farmer	40	11
Retired	18	4.9
Other	10	2.7
Residential area		
Rural	77	21.1
Urban	288	78.9

### Behavioral factors and dietary intake of type 2 DM patients

Out of 365 respondents, 161 (44.1%) and 12 (3.3%) of the study subjects were alcohol consumers and smokers respectively. About 247 (67.7%) of the study subjects had a habit of walking on their daily living and 302 (82.7%) had low level of vigorous physical activity. In our study 199 (55%) of the study participants had good dietary intake and 166 (45%) had poor dietary intake. Of these, 333 (91.2%) had got nutritional education from different sources and 32 (8.8%) individuals didn’t have nutritional education from any sources.

### Magnitude of overweight

The magnitude of overweight among type 2 DM patients using BMI as a measure was 149 (40.8%) [95% CI (35.7, 46)] and by using waist circumference as a measure 194 (53.2%) of the study subjects had central obesity (Table 2).

**Table 2:** Magnitude of overweight by socio-demographic characteristics among type 2 diabetes mellitus patients at Mekelle public hospitals, Tigray, Ethiopia, 2018

Variable	Category	Overweight	
		No	Yes
		Number (%)	Number (%)
Sex	Male	122 (56.5)	76 (51)
	Female	94 (43.5)	73 (49)
Age category	25–34	21 (9.7)	9 (6)
	35–44	61 (28.2)	10 (6.7)
	45–54	83 (38.4)	50 (33.6)
	Above 55	51 (23.6)	80 (53.7)
Marital status	Single	8 (3.7)	4 (2.7)
	Married	171 (79.2)	120 (80.5)
	Divorced	17 (7.9)	8 (5.4)
	Widowed	20 (9.3)	17 (11.4)
Religion	Orthodox	186 (86.1)	84 (56.4)
	Muslim	25 (11.6)	53 (35.6)
	Others	5 (2.3)	12 (8)
Ethnicity	Tigray	208 (96.3)	146 (98%)
	Amhara	8 (3.7)	3 (2)
Educational status	Can’t read and write	52 (24.1)	22 (14.8)
	Non formal education	1 (0.5)	0 (0.0)
	Primary school	56 (26)	38 (25.5)
	Secondary school	42 (19.4)	30 (20.1)
	Diploma and above	66 (30.6)	59 (39.6)
Occupational status	Housewife	48 (22.2)	39 (26.2)
	Government employee	86 (39.8)	41 (27.5)
	Merchant	34 (15.7)	49 (32.9)
	Farmer	35 (16.2)	5 (3.4)
	retried	5 (2.3)	13 (8.7)
	Other	8 (3.7)	2 (1.3)
Residential area	Rural	66 (30.6)	11 (7.4)
	Urban	150 (69.4)	138 (92.6)

### Factors associated with overweight

Being from urban residence had 3.4 times the odds of being overweight compared to their counterparts [AOR = 3.4, 95% CI (1.26–9.4)]. The odds of overweight among participants who stayed with type 2 DM for 3–6 years was 2.8 times higher compared to those who stayed less than 3 years [AOR = 2.8, 95% CI (1–7.85)]. Study participants who were alcohol consumers were 2.9 times more likely to develop overweight compared to non-consumers [AOR = 2.9, 95% CI (1.5–5.5)]. Overweight was 4 times higher among peoples with type 2 diabetes mellitus who had low level of vigorous physical activity as compared with study subjects who had adequate level of vigorous physical activity [AOR = 4, 95% CI (1.19–13.8)]. Besides, study subjects who did not walk regularly were 2.3 times more likely to have overweight as compared to their counterpart [AOR = 2.3, 95% CI (1–5.17)]. Concerning dietary intake, the odds of being overweight increased by nearly 8 times in type 2 DM patients with poor dietary intake [AOR = 7.9, 95% CI (4.02–15.5)] (Table 3).

**Table 3:** Factors associated with overweight among type 2 diabetes mellitus patients at Mekelle public hospitals, Tigray, Ethiopia, 2018

Variable	Category	Overweight		COR (95% CI)	AOR (95% CI)	p value
		No	Yes			
Age	25–34	21 (9.7)	9 (6)	1	1	
	35–44	61 (28.2)	10 (6.7)	0.4 (0.14, 1.07)	0.7 (0.18, 2.97)	0.658
	45–55	83 (38.4)	50 (33.6)	1.4 (0.6, 3.31)	1.4 (0.41, 5.05)	0.573
	> 55 years	51 (23.6)	80 (53.7)	3.66 (1.55–8.61)	3.5 (0.9, 12.3)	0.051
Residence area	Rural	66 (30.6)	11 (7.4)	1	1	
	Urban**	150 (69.4)	138 (92.6)	5.52 (2.8, 10.9)	3.4 (1.26, 9.4)	0.016
Alcohol drink	Yes**	68 (31.5)	93 (62.4)	3.61 (2.33–5.6)	2.9 (1.5, 5.5)	0.001
	No	148 (68.5)	56 (37.6)	1	1	
Alcohol drink frequency	7 days/week	6 (2.8%)	11 (7.4)	4.7 (1.67, 13.38)	1.2 (0.02, 15)	0.932
	5–6 days/week	10 (4.6%)	16 (10.7)	4.1 (1.76, 9.62)	0.2 (0.006, 8.03)	0.414
	1–4 days/week	17 (7.9)	23 (15.4)	3.4 (1.73, 7.00)	0.3 (0.01, 10.12)	0.518
	1 day/week	35 (16.2)	42 (28.2)	3 (1.79, 5.32)	0.2 (0.007, 5.9)	0.359
	Not drink	148 (69.5)	57 (38.3)	1	1	
Vigorous activity	Yes	85 (39.4)	24 (16.1)	1	1	
	No	131 (60.6)	125 (83.9)	3.37 (2, 5.65)	1.2 (0.48, 3.18)	0.656
Moderate activity	Yes	94 (43.5)	40 (26.8)	1	1	
	No	122 (56.5)	109 (73.2)	2.1 (1.33, 3.29)	0.7 (0.27, 1.66)	0.390
Walk	Yes	182 (84.3)	65 (43.6)	1	1	
	No**	34 (15.7)	84 (56.4)	6.9 (4.24, 11.27)	2.3 (1.01, 5.17)	0.045
Central obesity	Yes**	73 (33.80	121 (81.2)	8.46 (5.14, 13.9)	3.4 (1.64, 6.91)	0.001
	No	143 (66.2)	28 (18.8)	1	1	
Duration of DM	0–3 years	69 (31.9)	22 (14.8)	1	1	
	3–6 years**	46 (21.3)	32 (21.5)	2.1 (1.12, 4.21)	2.8 (1, 7.85)	0.039
	> 6 years	101 (46.8)	95 (63.8)	3 (1.69, 5.14)	2.5 (1.08, 5.74)	0.031
Dietary intake	Good	160 (74.1)	39 (26.2)	1	1	
	Poor**	56 (25.9)	110 (73.8)	8 (5.00, 12.96)	8 (4.02, 15.5)	0.000
Vigorous activity	Adequate	58 (26.9)	5 (3.4)	1	1	
	Low**	158 (73.1)	144 (96.6)	10.5 (4.12, 27)	4 (1.19, 13.8)	0.025
Moderate activity	Adequate	70 (32.4)	16 (10.7)	1	1	
	Low	146 (67.6)	133 (89.3)	3.98 (2.2, 7.20)	2.1 (0.89, 4.97)	0.089
Walk level	Adequate**	131 (60.6)	24 (16.1)	1	1	
	Low	85 (39.4)	125 (83.9)	8 (4.76, 13.43)	3.3 (1.45, 7.61)	0.005

*COR* Crude Odd Ratio, *AOR* Adjusted Odd Ratio, *CI* confidence interval

** p value < 0.05 significant

## Discussion

In this study, the overall magnitude of overweight was 40.8% [95% CI (35.7, 46)]. This is almost similar to the study done in Addis Ababa, and Hosanna [7, 8]. However, it is lower than the study done in India, Nepal and Bahrain [9–11]. This difference might be due to variation in socio-demographic factors, lifestyle, and economic status. In this study, using waist circumference as an indicator yielded the highest magnitude of obesity compared to using BMI. By using waist circumference as a measure 53.2% of the study subjects had central obesity that is higher as compare to the general obesity (40.8%). In general, this finding indicates that using BMI alone underestimate the magnitude of overweight or obesity.

Dietary intake was also the other variable that had significant association with overweight among type 2 DM patients. This is in line with the study done in Addis Ababa, Ethiopia [7]. It is also supported by study done in South Africa [12]. The reason might be due to rapid nutrition transition in developing countries and lack of nutritional education. There was also a statistically significant association between overweight and physical activities. This finding is in line with a study done in South Africa, Yemen, and Ghana where physical activity was significantly associated with overweight [12–14]. This might be due to high in sedentary behavior and poor motivation to physical exercise.

Residence area was the other variable that had a significant association with overweight in this study. Being from urban residence had 3.4 times odds of being overweight. The possible reason might be due exposure of urban population to unhealthy lifestyle, high proportion of urban study subjects in the current study and participants from rural area were more physically active. It is also showed that duration of diabetes after diagnosis was significantly associated with overweight. However, it contradicts with the finding reported from Yemen and Kenya which reports, the occurrence of overweight decreases with the duration of DM after diagnosis [13, 15]. So, this contradicting issue needs further investigation. Previous studies reported that being female associated with overweight however in this study sex had no significant association with overweight [9, 10]. This might be due to variation in socio-demographic factors.

## Conclusion and recommendations

The magnitude of overweight was high among type 2 DM patients. The determinant factors were residence area, alcohol consumption, low level physical activities, duration of DM, central obesity and dietary intake. Using waist circumference in conjunction with BMI would be useful for better diagnosis and early detection of overweight among type 2 DM patients. Researcher should have to investigate perceived barriers to regular physical activity among type 2 DM patients.

## Limitations of the study

- There might be recall bias among respondents answering questions related to dietary intake of the week, time spent for doing physical activity and sitting/reclining on a typical day.
- Nutritional knowledge and genetic susceptibility of the respondent to overweight was not considered.

## Data Availability

The datasets used and analyzed during this study are available from the corresponding author on reasonable request.

## Abbreviations

AOR: Adjusted Odds Ratio
BMI: body mass index
CI: confidence interval
COR: Crude Odds Ratio
DM: diabetes mellitus
SPSS: Statistical Package for Social Sciences
SSA: Sub-Saharan Africa
WC: waist circumference
